# Deterministic global optimization algorithm based on outer approximation for the parameter estimation of nonlinear dynamic biological systems

**DOI:** 10.1186/1471-2105-13-90

**Published:** 2012-05-10

**Authors:** Anton Miró, Carlos Pozo, Gonzalo Guillén-Gosálbez, Jose A Egea, Laureano Jiménez

**Affiliations:** 1 Departament d’Enginyeria Química, Universitat Rovira i Virgili, Tarragona, Spain; 2 Departamento de Matemática Aplicada y Estadística, Universidad Politécnica de Cartagena, Cartagena, Spain

## Abstract

**Background:**

The estimation of parameter values for mathematical models of biological systems is an optimization problem that is particularly challenging due to the nonlinearities involved. One major difficulty is the existence of multiple minima in which standard optimization methods may fall during the search. Deterministic global optimization methods overcome this limitation, ensuring convergence to the global optimum within a desired tolerance. Global optimization techniques are usually classified into stochastic and deterministic. The former typically lead to lower CPU times but offer no guarantee of convergence to the global minimum in a finite number of iterations. In contrast, deterministic methods provide solutions of a given quality (i.e., optimality gap), but tend to lead to large computational burdens.

**Results:**

This work presents a deterministic outer approximation-based algorithm for the global optimization of dynamic problems arising in the parameter estimation of models of biological systems. Our approach, which offers a theoretical guarantee of convergence to global minimum, is based on reformulating the set of ordinary differential equations into an equivalent set of algebraic equations through the use of orthogonal collocation methods, giving rise to a nonconvex nonlinear programming (NLP) problem. This nonconvex NLP is decomposed into two hierarchical levels: a master mixed-integer linear programming problem (MILP) that provides a rigorous lower bound on the optimal solution, and a reduced-space slave NLP that yields an upper bound. The algorithm iterates between these two levels until a termination criterion is satisfied.

**Conclusion:**

The capabilities of our approach were tested in two benchmark problems, in which the performance of our algorithm was compared with that of the commercial global optimization package BARON. The proposed strategy produced near optimal solutions (i.e., within a desired tolerance) in a fraction of the CPU time required by BARON.

## Background

Elucidation of biological systems has gained wider interest in the last decade. Despite recent advances, fundamental understanding of life processes still requires powerful theoretical tools from mathematics and physical sciences. Particularly, mathematical modelling of biological systems is nowadays becoming an essential partner of experimental work. One of the most challenging tasks in computational modelling of biological systems is the estimation of the model parameters. The aim here is to obtain the set of parameter values that make the model response consistent with the data observed. Parameter estimation can be formulated as an optimization problem in which the sum of squared residuals between the measured and simulated data is minimized. The biological model dictates the type of optimization problem being faced. Many biological systems are described through nonlinear ordinary differential equations (ODEs) that provide the concentration profiles of certain metabolites over time. Recent methodological developments have enabled the generation of some dynamic profiles of gene networks and protein expression data, although the latter are still very rare. In this context, there is a strong motivation for developing systematic techniques for building dynamic biological models from experimental data. The parameter estimation of these models gives rise to dynamic optimization problems which are hard to solve.

Existing approaches to optimize dynamic models can be roughly classified as direct or indirect (also known as variational) [1]. Direct methods make use of gradient-based nonlinear programming (NLP) solvers and can in turn be divided into sequential and simultaneous. In sequential approaches, the optimization of the control variables, which are discretized, is performed by a NLP solver, whereas the ODE is calculated externally, that is, both steps are executed in a sequential manner. In contrast, in simultaneous strategies, both the control and state profiles are approximated using polynomials (e.g., Lagrange polynomials) and discretized in time by means of finite elements [2,3]. In the latter strategy, the ODE system is replaced by a system of algebraic equations that is optimized with a standard gradient-based NLP solver. Simultaneous approaches can handle dynamic systems with unstable modes and with path constraints [1]. Furthermore, they allow performing automatic differentiation with respect to the control and state variables, avoiding the need to calculate the derivatives numerically as is the case in the sequential approach. Unfortunately, the discretization step can lead to large scale NLPs that are difficult to solve.

Models of biological systems are typically highly nonlinear, which gives rise to nonconvex optimization problems with multiple local solutions (i.e., multimodality). Because of this, traditional gradient-based methods used in the sequential and simultaneous approaches may fall in local optima. In the context of parameter estimation, these local solutions should be avoided, since they may lead to inaccurate models that are unable to predict the system’s performance precisely.

Global optimization (GO) algorithms are a special class of techniques that attempt to identify the global optimum in nonconvex problems. These methods can be classified as stochastic and deterministic. Stochastic GO methods are based on probabilistic algorithms that provide near optimal solutions in short CPU times. Despite having shown great potential with large-scale problems like parameter estimation [4], these methods have as major limitation that are unable to guarantee convergence to the global optimum in a finite number of iterations. In other words, they provide solutions whose optimality (i.e., quality) is unknown, and may or may not be globally optimal. In contrast, deterministic global optimization methods ensure global optimality within a desired tolerance, but lead to larger computational burdens. Hence, in addition to the solution itself, these methods provide as output a rigorous interval within which the best possible solution (i.e., global optimum) must fall. Despite recent advances in deterministic global optimization methods [5,6], their application to parameter estimation has been quite scarce. Two main deterministic GO methods exist: spatial branch-and-bound (sBB) [2,5-7], and outer approximation [8]. Both algorithms rely on computing valid lower and upper bounds on the global optimum. These bounds tend to approach as iterations proceed, thus offering a theoretical guarantee of convergence to the global optimum.

A rigorous lower bound on the global optimum of the original nonconvex problem is obtained by solving a valid relaxation that contains its feasible space. To construct this relaxed problem, the nonconvex terms in the original formulation are replaced by convex envelopes that overestimate its feasible region. There are different types of convex envelopes that provide relaxations for a wide variety of nonconvexities. These relaxations are the main ingredient of deterministic GO methods and play a key role in their performance. In general, tighter relaxations provide better bounds (i.e., closer to the global optimum), thereby expediting the overall solution procedure.

To the best of our knowledge, Esposito and Floudas were the first to propose a deterministic method for the global solution of dynamic optimization problems with embedded ODEs [2]. Their approach relies on reformulating the problem as a nonconvex NLP using orthogonal collocation on finite elements. This reformulated NLP was then solved by means of a sBB method. To this end, they constructed a convex relaxation of the reformulated problem following the *α*BB approach previously proposed by the authors [5-7]. Despite being valid for twice continuous differentiable functions, these relaxations may provide weak bounds in some particular cases and therefore lead to large CPU times when used in the context of a spatial branch and bound framework [9].

This work proposes a computational framework for the deterministic global optimization of parameter estimation problems of nonlinear dynamic biological systems. The main contributions of our work are: (1) the application of deterministic global optimization methods to dynamic models of biological systems, and (2) the use of several known techniques employed in dynamic (i.e., orthogonal collocation on finite elements) and global optimization (i.e., symbolic reformulation of NLPs and piecewise McCormick envelopes) in the context of an outer approximation algorithm. The approach presented relies on discretizing the set of nonlinear ODEs using orthogonal collocation on finite elements, thereby transforming the dynamic system into an equivalent nonconvex NLP problem. A customized outer approximation algorithm that relies on a mixed-integer linear programming (MILP) relaxation is used in an iterative scheme along with the aforementioned NLP to solve the nonconvex model to global optimality. The MILP relaxation is tightened using a special type of cutting plane that exploits the problem structure, thereby expediting the overall solution procedure.

The capabilities of our algorithm are tested through its application to two case studies: the isomerisation of *α*-Pinene (case study 1) and the inhibition of HIV proteinase (case study 2). The results obtained are compared with those produced by the state-of-art commercial global optimization package BARON (Branch And Reduce Optimization Navigator). Our algorithm is proved from these numerical examples to produce near optimal solutions in a fraction of the CPU time required by BARON.

## Methods

### Problem statement

The problem addressed in this work can be stated as follows: given is a dynamic kinetic model describing the mechanism of a set of biochemical reactions. The goal is to determine the appropriate values of the model coefficients (e.g., rate constants, initial conditions, etc.), so as to minimize the sum-of-squares of the residuals between the simulated data provided by the model and the experimental observations.

### Mathematical formulation

We consider dynamic parameter estimation optimization problems of the following form:

(1) $$\underset{\theta ,{\hat{z}}_{u}}{\min}\;\underset{j\in J\;M}{\sum }\underset{u\in U}{\sum }{\left({\hat{z}}_{u,\;j}-{\bar{z}}_{u,\;j}\right)}^{2}$$

(2) $$\mathrm{s.t.}\;{\dot{z}}_{j}=g(z,\theta ,t)\;\forall j\in J$$

(3) $$z_{j}(t_{0})=z_{0}\;\;\;\;\;\forall j\in J$$

(4) $$t\in \;\;[t_{0},t_{f}]$$

(5) $${\hat{z}}_{u,\;j}=z_{j}(t_{u})\;\;\forall u\in U;\;\forall j\in J\;M$$

Where $\dot{z}$ represents the state variables (i.e., metabolite concentrations), ***z***_0_ their initial conditions, ${\hat{z}}_{u,\;j}$ represents the experimental data variables, ${\bar{z}}_{u,\;j}$ are the experimental observations, *J* is the set of state variables whose derivatives explicitly appear in the model, ***θ*** are the parameters to be estimated and *t*_*u*_, is the time associated with the *u*th experimental data point in the set *U*.

**Figure 1:**
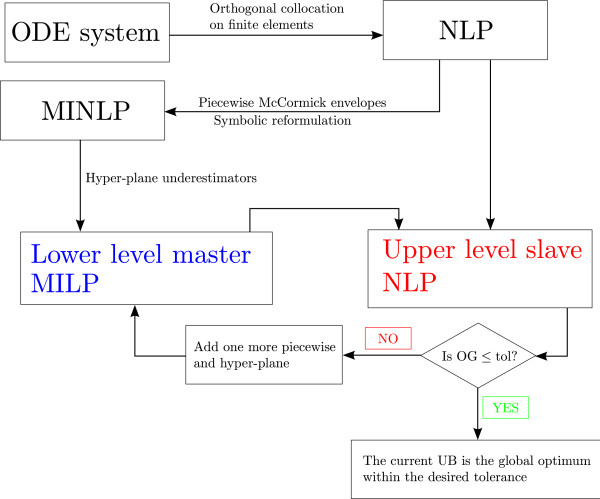
**Solution Strategy.** The system of ODEs is first reformulated into a nonconvex NLP using the orthogonal collocation on finite elements approach. This NLP is decomposed into two levels: a master MILP and a slave NLP. The master MILP, which is constructed using piecewise McCormick envelopes and supporting hyper-planes, provides a rigorous lower bound on the global optimum. The slave NLP corresponds to the original nonconvex NLP that is solved using as starting point the solution of the MILP. The algorithm iterates between these two levels until the optimality gap (i.e., the relative difference between the upper and lower bounds) is reduced below a given tolerance.

Our solution strategy relies on reformulating the nonlinear dynamic optimization problem as a finite-dimensional NLP by applying a complete discretization using orthogonal collocation on finite elements. This NLP is next solved using an outer approximation algorithm (see Figure 1). In the sections that follow, we explain in detail the main steps of our algorithm.

#### Orthogonal collocation approach

There is a considerable number of collocation-based discretizations for the solution of differential-algebraic systems [10]. Without loss of generality, we employ herein the so-called orthogonal collocation on finite elements method [11,12]. Consider the following set of ODE’s defined as

(6) $${\dot{z}}_{j}=g(z,\theta ,t)\;\forall j\in J$$

The state variables are first approximated using Lagrange polynomials as follows:

(7) $$z_{N\;K+1}(t)=\overset{N\;K}{\underset{k=0}{\sum }}\xi_{k}\phi_{k}(t)\;\phi_{k}(t)=\overset{N\;K}{\underset{q=0,\;q\neq k}{\prod }}\frac{t-t_{q}}{t_{k}-t_{q}}$$

These polynomials have the property that at the orthogonal collocation points their coefficients, *ξ*_*k*_, take the value of the state profile at that point. Therefore, the collocation coefficients *ξ*_*k*_ acquire physical meaning which allows to generate bounds for these variables.

**Figure 2:**
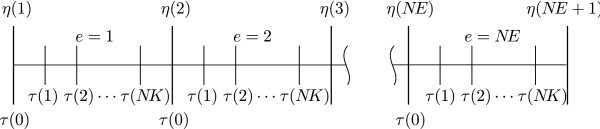
**Orthogonal collocation discretization over finite elements.** The time interval is divided into *NE* elements which in turn are divided into *NK* + 1 collocation points evaluated at the shifted orthogonal Legendre polynomials.

Because state variables may present steep variations, the whole solution space is commonly divided into time intervals called finite elements. Hence, the time variable *t* is divided into *NE* elements of length Δ *η*_*e*_ and rescaled as *τ*∈[0,1]. Within each finite element, *NK* + 1 orthogonal collocation points *τ*(0), *τ*(1), *τ*(2), ⋯ ,*τ*(*NK*) are distributed at the shifted (between 0 and 1) roots of the orthogonal Legendre polynomial of *NK* degree. Recall that the 0th orthogonal collocation point is located at the beginning of each element (Figure 2).

Following the collocation method [10], the residual equations arising from the combination of Eqs. 6 and 7, are defined for each element *e* in the set *E* and state variable in the set *J*, giving rise to the following constraints:

(8) $$\begin{array}{ll} \overset{N\;K}{\underset{k=0}{\sum }}\xi_{e,\;k,\;j}{\dot{\phi }}_{e,\;k,\;j}(\tau_{k^{\prime }})-\Delta \eta_{e}g_{j}(\xi_{e,\;k^{\prime },\;j},\;\theta ,\;t_{e,\;k^{\prime }})=0 & \;\\ \forall e\in E\;k^{\prime }=1,\;\ldots ,\;N\;K;\;\forall j\in J & \; \end{array}$$

The state variables have to be continuous between elements, so we enforce the following continuity constrains:

(9) $$\xi_{e,\;0,\;j}\;-\;\overset{N\;K}{\underset{k=0}{\sum }}\xi_{e-1,\;k,\;j}\phi_{k}(\tau \;=\;1)\;=\;0\;e\;=\;2,\;\ldots ,\;N\;E\;\forall j\in J$$

These equations extrapolate the polynomial at element *e*-1, providing an accurate initial condition for the next element *e*.

Moreover, initial conditions are enforced for the beginning of the first element using the following equation:

(10) $$\xi_{1,\;0,\;j}-z_{0,\;j}=0\;\forall j\in J$$

Recall that collocation points in which time has been discretized will not necessarily match the times at which experimental profiles were registered. Hence, variable ${\hat{z}}_{u,j}$ is added to determine the value of the model states profiles at times *t*_*u*_ making it possible to fit the model to the experimental points. This is accomplished by adding the following equation:

(11) $$-{\hat{z}}_{u,\;j}+\overset{N\;K}{\underset{k=0}{\sum }}\xi_{e_{u},\;k,\;j}\phi_{k}(\tau_{u})=0\;\forall u\in U;\;\forall j\in J\;M$$

Where *τ*_*u*_ is calculated as follows:

(12) $$\tau_{u}=\frac{t_{u}-\eta_{e_{u}}}{\Delta \eta_{e_{u}}}$$

Here, the subscript *e*_*u*_ refers to the element where *t*_*u*_ falls, that is, *e*_*u*_≡{*e*:*η*_*e*_≤*t*_*u*_<*η*_*e* + 1_}.

#### NPL formulation

The dynamic optimization problem is finally reformulated into the following NLP:

(13) $$\underset{\theta ,\;\xi ,\;{\hat{z}}_{u}}{\min}\;\;\;\;\;\underset{j\in J\;M}{\sum }\underset{u\in U}{\sum }{\left({\hat{z}}_{u,\;j}-{\bar{z}}_{u,\;j}\right)}^{2}$$

(14) $$\begin{array}{l} \mathrm{s.t.}\;\;\overset{N\;K}{\underset{k=0}{\sum }}\xi_{e,\;k,\;j}{\dot{\phi }}_{e,\;k,\;j}(\tau_{k^{\prime }})-\Delta \eta_{e}g_{j}(\xi_{e,\;k^{\prime },\;j},\;\theta ,\;t_{e,\;k^{\prime }})\;=\;0\\ \;\;\;\forall e\in E\;k^{\prime }=1,\;\ldots ,\;N\;K;\;\forall j\in J \end{array}$$

(15) $$\begin{array}{l} \xi_{e,\;0,\;j}-\overset{N\;K}{\underset{k=0}{\sum }}\xi_{e-1,\;k,\;j}\phi_{k}(\tau =1)=0\\ e=2,\;\ldots ,\;N\;E\;\forall j\in J \end{array}$$

(16) $$\xi_{1,\;0,\;j}-z_{0,\;j}=0\;\forall j\in J$$

(17) $$\begin{array}{l} -{\hat{z}}_{u,\;j}+\overset{N\;K}{\underset{k=0}{\sum }}\xi_{e_{u},\;k,\;j}\phi_{k}(\tau_{u})=0\\ \forall u\in U;\;\forall j\in J\;M \end{array}$$

## Results and discussion

### Optimization approach

**Figure 3:**
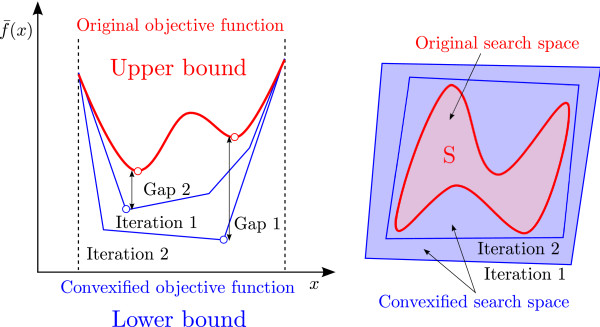
**Optimization algorithm based on outer approximation.** Our approach decomposes the problem into two subproblems: a master MILP, constructed by relaxing the original model using piecewise McCormick envelopes and hyper-planes, that provides a lower bound, and a slave NLP that yields an upper bound. The algorithm iterates between these two levels until a termination criterion is satisfied.

The method devised for globally optimizing the NLP that arises from the reformulation of the parameter estimation problem (Eqs. 1317) is based on an outer approximation algorithm [8] used by the authors in previous works [13-17]. This approach relies on decomposing the original NLP into two subproblems at different hierarchical levels: a lower level MILP problem and an upper level slave NLP problem. The master problem is a relaxation of the original NLP (i.e., it overestimates its feasible region) and hence provides a rigorous lower bound on its global optimum. The slave NLP yields a valid upper bound when it is solved locally. The algorithm iterates between these two levels until the optimality gap (i.e., the relative difference between the upper and lower bounds) is reduced below a given tolerance (Figure 3). In the following subsections, we provide a detailed description of the algorithm.

#### Lower level master problem

Designing efficient and smart strategies for attaining tight bounds is a mayor challenge in deterministic global optimization. Both the quality of the bounds and the time required to generate them drastically influence the overall performance of a deterministic global optimization algorithm.

Any feasible solution of the original NLP is a valid upper bound and can be obtained by means of a local NLP solver. To obtain lower bounds, we require a rigorous convex (linear or nonlinear) relaxation. This relaxation is obtained by replacing the nonconvex terms by convex overestimators. Since the relaxed problem is convex, it is possible to solve it to global optimality using standard local optimizers. Furthermore, since its feasible region contains that of the original problem and its objective function rigorously underestimates the original one, it is guaranteed to provide a lower bound on the global optimum of the original nonconvex model [18].

Androulakis et al. [19] proposed a convex quadratic relaxation for nonconvex functions named *α*BB underestimator which can be applied to general twice continuously differentiable functions. This technique, which was used in parameter estimation by Esposito and Floudas [2], might lead in some cases to weak relaxations and therefore poor numerical performance [9].

To construct a valid MILP relaxation, we apply the following approach. We first reformulate the NLP using the symbolic reformulation method proposed by Smith and Pantelides [20]. This technique reformulates any system of nonlinear equations into an equivalent canonical form with the only nonlinearities being bilinear products, linear fractional, simple exponentiation and univariate function terms with the following standard form:

(18) $$\underset{w}{\min}\;\;\;\;\;\;\;\;\;\;w_{\mathrm{obj}}$$

(19) s.t.Aw=b

(20) $$w^{l}\leq \;w\leq w^{u}$$

(21) $$y\in [y^{l},\;\ldots ,\;y^{u}]$$

(22) $$w_{k}\equiv w_{i}w_{j}\;\forall (i,\;j,\;k)\in T_{\mathrm{bt}}$$

(23) $$w_{k}\equiv \frac{w_{i}}{w_{j}}\;\;\;\;\;\forall (i,\;j,\;k)\in T_{\mathrm{lft}}$$

(24) $$w_{k}\equiv w_{i}^{n}\;\;\;\;\;\forall (i,\;k,\;n)\in T_{\mathrm{et}}$$

(25) $$w_{k}\equiv \mathrm{fn}(w_{i})\;\;\;\forall (i,\;k)\in T_{\mathrm{uft}}$$

where vector *w* comprises continuous variables *x* as well as integers *y*, while the sets $T_{\mathrm{bt}}$, $T_{\mathrm{lft}}$, $T_{\mathrm{et}}$ and $T_{\mathrm{uft}}$ are the bilinear product, linear fractional, simple exponentiation and univariate function terms, respectively.

A rigorous relaxation of the original model is constructed by replacing the nonconvex terms in the reformulated model by convex estimators. The solution of the convex relaxation provides a valid lower bound on the global optimum. More precisely, the bilinear terms are replaced by piecewise McCormick relaxations. The fractional terms can be convexified in two different manners. The first is to replace them by tailored convex envelopes that exploit their structure [21]. The second is to express them as bilinear terms by performing a simple algebraic transformation, and then use the McCormick envelopes to relax the associated bilinear term. Univariate functions commonly used in process engineering models (e.g., logarithms, exponentials, and square roots) are purely convex or purely concave, and can be replaced by the exact function-secant pair estimators [22].

The reader is referred to the work by Smith and Pantelides [20] for further details on the symbolic reformulation. We focus next on explaining how the bilinear terms are approximated in the reformulated NLP.

##### Piecewise McCormick-based relaxation

The bilinear terms appearing in the reformulated model are approximated using McCormick’s envelopes [23-26]. For bilinear terms, this relaxation is tighter than the *α*BB-based relaxations [18,27].

Each bilinear term *xy* can be replaced by an auxiliary variable *z* as follows:

(26) $$z=\mathrm{xy}\;x^{L}\leq x\leq x^{U}\;y^{L}\leq y\leq y^{U}$$

The best known relaxation for approximating a bilinear term is given by the McCormick envelopes, obtained by replacing Eq. 26 by the following linear under (Eqs. 27 and 28), and overestimators (Eqs. 29 and 30):

(27) $$z\geq xy^{L}+x^{L}y-x^{L}y^{L}$$

(28) $$z\geq xy^{U}+x^{U}y-x^{U}y^{U}$$

(29) $$z\leq xy^{L}+x^{U}y-x^{U}y^{L}$$

(30) $$z\leq xy^{U}+x^{L}y-x^{L}y^{U}$$

In this work we further tighten the McCormick envelopes by adding binary variables [25,28]. Particularly, two additional sets of variables are defined in the piecewise formulation:

Binary switch: $\left(\lambda \in {\left\{0,\;1\right\}}^{N_{P}}\right)$

Continuous switch: $\left(\Delta y\in {\left[0,\;y^{U}-y^{L}\right]}^{N_{P}}\right)$

**Figure 4:**
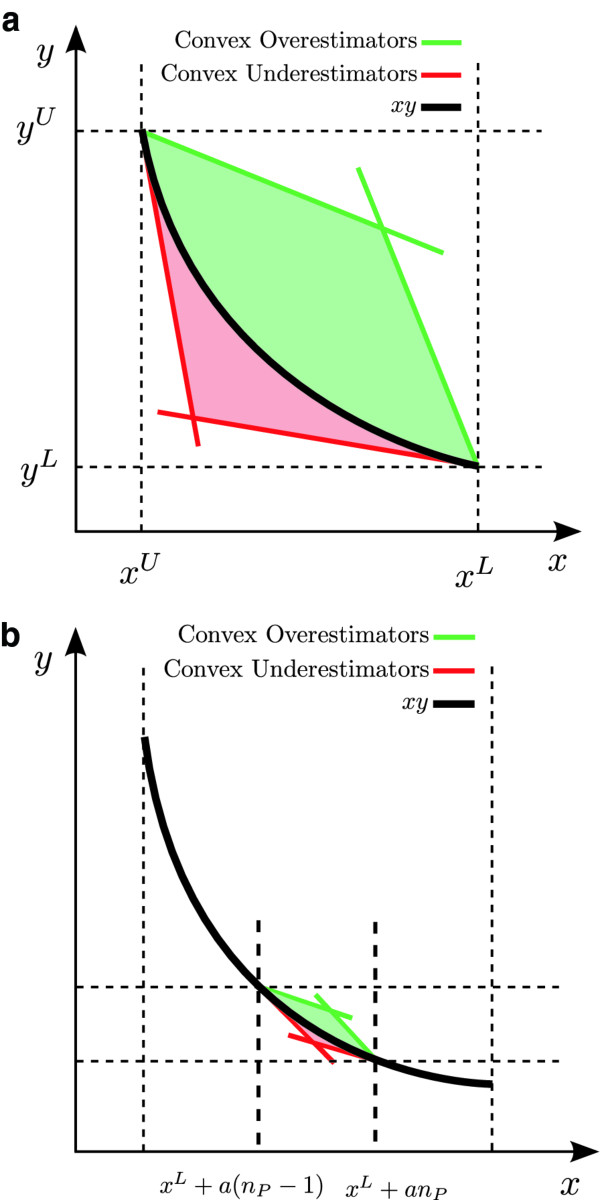
**McCormick convex relaxation over the entire feasible region (subfigure (a)) compared to a piecewise McCormick relaxation over a smaller active region (subfigure (b)) where the tightness of the relaxation is improved.** We built the master problem by replacing the bilinear terms by piecewise McCormick envelopes. The relaxation can be further improved by adding binary variables.

The binary switch *λ* is active (i.e., *λ*(*n*_*P*_)=1) for the segment where *x* is located $\left(x^{L}+a(n_{P}-1)\leq x\leq x^{L}+an_{P}\right)$ and is otherwise inactive. Therefore, the partitioning scheme activates exactly only one *n*_*P*_∈{1,…,*N*_*P*_} so that the feasible region corresponding to the relaxation of *xy* is reduced from the parallelogram in Figure 4(a) to a significantly smaller one depicted in Figure 4(b).

Eq. 31 enforces that only one binary variable is active:

(31) $$\overset{N_{P}}{\underset{n_{P}=1}{\sum }}\lambda (n_{P})=1$$

The continuous switch Δ*y* takes on any positive value between 0 and *y*^*U*^−*y*^*L*^ when the binary switch corresponding to the *n*_*P*_th piecewise *λ*(*n*_*P*_) is active (i.e., *λ*(*n*_*P*_)=1) and 0 otherwise. Therefore:

(32) $$y=y^{L}+\overset{N_{P}}{\underset{n_{P}=1}{\sum }}\Delta y(n_{P})$$

(33) $$0\leq \Delta y(n_{P})\;\leq \;(y^{U}-y^{L})\lambda (n_{P})\;n_{P}\;=\;1,\ldots ,\;N_{P}$$

Finally, the under and overestimators for the active segment are defined in algebraic terms as follows:

(34) $$z\geq xy^{L}+\overset{N_{P}}{\underset{n_{P}=1}{\sum }}[x^{L}+a(n_{P}-1)]\Delta y(n_{P})$$

(35) $$z\geq xy^{U}+\overset{N_{P}}{\underset{n_{P}=1}{\sum }}[x^{L}+an_{P}][\Delta y(n_{P})-(y^{U}-y^{L})\lambda (n_{P})]$$

(36) $$z\leq xy^{L}+\overset{N_{P}}{\underset{n_{P}=1}{\sum }}[x^{L}+an_{P}]\Delta y(n_{P})$$

(37) $$\begin{array}{cc} z & \leq xy^{U}\;+\;\;\overset{N_{P}}{\underset{n_{P}=1}{\sum }}[x^{L}+a(n_{P}-1)][\Delta y(n_{P})\;-\;(y^{U}-y^{L})\\ \;\times \lambda (n_{P})] \end{array}$$

(38) $$x^{L}\leq x\leq x^{U};\;y^{L}\leq y\leq y^{U}$$

Note that the discrete relaxation is tighter than the continuous one over the entire feasible region. The introduction of the binary variables required in the piecewise McCormick reformulation gives rise to a mixed-integer nonlinear programming (MINLP) problem, with the only nonlinearities appearing in the objective function. While this MINLP is convex and can be easily solved to global optimality with standard MINLP solvers, it is more convenient to linearize it in order to obtain an MILP formulation, for which more efficient software packages exist. The section that follows explains how this is accomplished.

##### Hyper-planes underestimation

**Figure 5:**
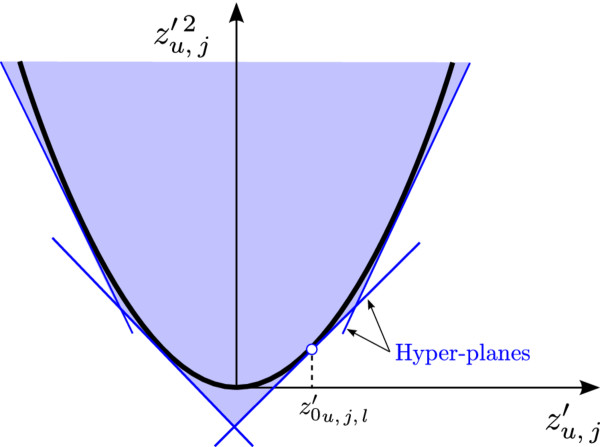
***x *squared function underestimated by a 1st degree Taylor series.** The objective function is linearized by a first degree Taylor series with *l* hyper-planes.

The convex MINLP can be further reformulated into an MILP by replacing the objective function by a set of hyper-planes. For this, we define two new variables as $z_{u,\;j}^{\prime }={\hat{z}}_{u,\;j}-{\bar{z}}_{u,\;j}$ and $\alpha \geq {z^{\prime }}_{u,\;j}^{\;2}$. The quadratic terms are then approximated by 1st degree Taylor series. That is, the square terms are replaced by *l* hyper-planes uniformly distributed between the maximum and minimum desired values of $z_{u,\;j}^{\prime }$ (Figure 5) so that the objective function is reduced to a summation of quadratic terms as follows:

(39) $$\underset{\theta ,\;\xi ,\;{\hat{z}}_{u}}{\min}\;\;\;\;\;\;\;\underset{j\in J\;M}{\sum }\underset{u\in U}{\sum }\alpha_{u,\;j}$$

(40) $$\begin{array}{cc} \;\; & \;\;\alpha_{u,\;j}\geq {{z^{\prime }}_{0}^{\;2}}_{u,\;j,\;l}+2{z_{0}^{\prime }}_{u,\;j,\;l}(z_{u,\;j}^{\prime }-{z_{0}^{\prime }}_{u,\;j,\;l})\\ \;\;\;\;\forall u\in U\;\forall j\in J\;M\;\forall l\in L \end{array}$$

#### Upper level slave problem

A valid upper bound on the global optimum is obtained by optimizing the original NLP locally. This NLP is initialized using the solution provided by the MILP as starting point. The solution of this NLP is used to tighten the MILP, so the lower and upper bounds tend to converge as iterations proceed.

#### Algorithm steps

The proposed algorithm comprises the following steps:

1. Set iteration count it = 0, UB = *∞*, LB = −*∞* and tolerance error = tol.

2. Set it=it + 1. Solve the master problem MILP.

(a) If the MILP is infeasible, stop (since the NLP is also infeasible).

(b) Otherwise, update the current LB making $\mathrm{LB}=\underset{\mathrm{it}}{\max}({\mathrm{LB}}_{\mathrm{it}})$, where LB_it_ is the value of the objective function of the MILP in the it^th^ iteration.

3. Solve the slave problem NLP.

(a) If the NLP is infeasible add one more piecewise term and hyper-plane to the master MILP and go to step 2 of the algorithm.

(b) Otherwise, update the current UB making UB = $\underset{\mathrm{it}}{\min}$ (UB_it_), where UB_it_ is the value of the objective function of the NLP in the it^th^ iteration.

4. Calculate the optimality gap OG as $\mathrm{OG}=\frac{\left|\mathrm{UB}-\mathrm{LB}\right|}{\mathrm{UB}}$.

(a) If OG≤tol, then stop. The current UB is regarded as the global optimum within the desired tolerance.

(b) Otherwise, add one more piecewise section and hyper-plane to the master MILP and go to step 2 of the algorithm.

Remarks:

There are different methods to update the piecewise bilinear approximation. One possible strategy is to update it by dividing the active piecewise (i.e., the piecewise term in which the solution is located) into two equal-length segments.

The new hyper-plane term ${z_{0}^{\prime }}_{u,\;j,\;l}$ is added at the optimal solution of the MILP (solution point $z_{u,\;j}^{\prime }$) in the previous iteration.

The univariate convex and concave terms in the reformulated problem can be either approximated by the secant or by a piecewise univariate function similarly as done with the McCormick envelopes.

Our algorithm needs to be tuned prior to its application. This is a common practice in any optimization algorithm. In a previous publication [13], we studied the issue of defining the number of piecewise intervals and supporting hyper-planes in an optimal manner. In practice, however, the optimal number of piecewise terms and hyper-planes is highly dependent on the specific instance being solved, so it is difficult to provide general guidelines on this.

The approach presented might lead to large computational burdens in large-scale models of complex biological systems. Future work will focus on expediting our algorithm through the addition of cutting planes and the use of customized decomposition strategies.

### Case studies

We illustrate the performance of the proposed algorithm through its application to two challenging benchmark parameter estimation problems: the isomerisation of *α*-Pinene (case study 1) and the inhibition of HIV proteinase (case study 2). The objective in these problems is to obtain the set of values of the model parameters such that the model response is as close as possible to the experimental data. For comparison purposes we used the global optimization package BARON (version 8.1.5). BARON is a commercial software for solving nonconvex optimization problems to global optimality. BARON combines constraint propagation, interval analysis, duality, and enhanced "branch and bound" concepts for efficient range reduction with rigorous relaxations constructed by enlarging the feasible region and/or underestimating the objective function. The interested readers have the possibility to evaluate this software on their own for free in this link: http://www.neos-server.org/neos/solvers/go:BARON/GAMS.html. Our algorithm was implemented in GAMS 23.5.2 using CPLEX 12.2.0.0 for the MILPs and SNOPT 4 for the NLPs subproblems. All the calculations were performed in a PC/AMD Athlon II at 2.99 Ghz using a single core. Data about the size of the models can be found in Table 1.

**Table 1:** Model size in the last iteration

	**Isomerisation of *α*-Pinene**	**Inhibition of HIV proteinase**
MILP equations	1,836	138,128
MILP continuous variables	1,096	53,321
MILP binary variables	380	3,625
NLP equations	186	16,306
NLP variables	196	16,361

#### Case study 1: Isomerisation of *α*-Pinene

**Figure 6:**
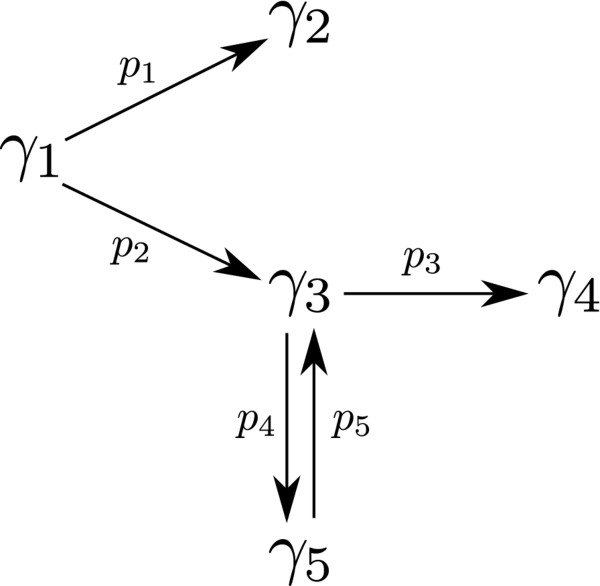
**Proposed mechanism describing the thermal isomerization of ***α***-Pinene.** In this reaction *α*-Pinene (*γ*_1_) is thermally isomerized to dipentene (*γ*_2_) and allo-ocimene (*γ*_3_), which in turn yields *α*- and *β*-Pyronene (*γ*_4_) and a dimer (*γ*_5_).

In this first case study, five kinetic parameters describing the thermal isomerisation of *α*-Pinene are estimated. The proposed reaction scheme for this process is depicted in Figure 6. In this homogeneous chemical reaction, *α*-Pinene (*γ*_1_) is thermally isomerised to dipentene (*γ*_2_) and allo-ocimene (*γ*_3_), which in turn yields *α*- and *β*-Pyronene (*γ*_4_) and a dimer (*γ*_5_). This process was originally studied by Fuguitt and Hawkins [29], which carried out a single experiment reporting the experimental concentrations (mass fraction) of the reactant and the four products measured at eight time intervals.

Hunter and McGregor [30] postulated first-order kinetics and proposed the following set of ODE’s describing the dynamic process:

(41) $$\frac{d\gamma_{1}}{\mathrm{dt}}=-(p_{1}+p_{2})\gamma_{1}$$

(42) $$\frac{d\gamma_{2}}{\mathrm{dt}}=p_{1}\gamma_{1}$$

(43) $$\frac{d\gamma_{3}}{\mathrm{dt}}=p_{2}\gamma_{1}-(p_{3}+p_{4})\gamma_{3}+p_{5}\gamma_{5}$$

(44) $$\frac{d\gamma_{4}}{\mathrm{dt}}=p_{3}\gamma_{3}$$

(45) $$\frac{d\gamma_{5}}{\mathrm{dt}}=p_{4}\gamma_{3}-p_{5}\gamma_{5}$$

(46) $$\gamma_{0}=\;[100,\;0,\;0,\;0,\;0]\;t\in [0,\;36420]$$

Rodriguez-Fernandez et al. [4] addressed this problem by applying a metaheuristic based on the scatter search method. This strategy does not offer any theoretical guarantee of convergence to the global optimum in a finite number of iterations.

Following our approach, the state variables were approximated by Lagrange polynomials using three collocation points evaluated at the shifted roots of orthogonal Legendre polynomials and defining five finite elements of equal length. The nonconvexities in the resulting residual equations are given by the bilinear terms *θ*_*i*_*ξ*_*e*,*k*, *j*_ which were relaxed using piecewise McCormick approximations as described previously. The objective function was underestimated using supporting hyper-planes.

It is well known that the quality of the lower bound predicted by a relaxation strongly depends on the bounds imposed on its variables [31]. Hence bounds on collocation coefficients ($\xi_{e,\;k,\;j}^{L}$ and $\xi_{e,\;k,\;j}^{U}$, originally set to 0 and 100, respectively) were tightened by performing a bound contraction procedure [21,32]. Particularly, tight lower and upper bounds were estimated for each collocation coefficient by maximizing and minimizing its value while satisfying the constraints contained in the master problem. This is a costly process (i.e., if bounds for *n* variables are to be estimated, *2n* optimization problems should be solved). For this reason, it was only performed recursively 3 times before the initialization of the algorithm. The MILP was further tightened by adding the following constraint:

(47) $$\underset{j\in J\;M}{\sum }\underset{u\in U}{\sum }{\left({\hat{z}}_{u,\;j}-{\bar{z}}_{u,\;j}\right)}^{2}\leq 20$$

which forces the model to find a solution better than the one obtained at the beginning of the search by locally minimizing the original NLP (i.e., 20 is a rigorous upper bound for the objective function). Furthermore, the parameter *θ*_*i*_ was allowed to take any value within the [0, 1] interval.

The problem was solved with 6 initial hyper-planes. An extra hyper-plane was added in each iteration, but the total number of piecewise terms was kept constant (4 piecewise intervals were considered) in order to keep the MILP in a manageable size. A tolerance of 5% was set as termination criterion.

For comparison purposes, we solved the same problem with the standard global optimization package BARON using its default settings. BARON was able to find the global optimum but failed at reducing the optimality gap below the specified tolerance after 12h of CPU time. In contrast, our algorithm closed the gap in less than 3h (see Table 2). As shown in Table 2, the results obtained agree with those reported in the literature.

**Table 2:** **Global optimization results for the *α*-Pinene isomerisation problem**

	**Rodriguez-Fernandez et al.**	**BARON**	**Proposed algorithm**
Sum of squares	19.87	19.87	19.87
UB	-	19.87	19.87
LB	-	4.112	19.26
Gap (%)	-	79.31	3.056
Iterations	9,518	60,614	2
Time (CPU s)	122	43,200	8,916

#### Case study 2: Inhibition of HIV proteinase

**Figure 7:**
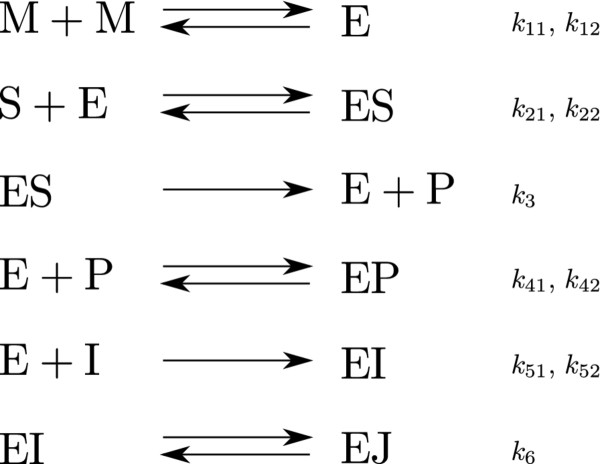
**Proposed mechanism describing the irreversible inhibition of HIV proteinase.** The enzyme HIV proteinase (E), which is only active in a dimer form, was added to a solution of an irreversible inhibitor (I) and a fluorogenic substrate (S). The product (P) is a competitive inhibitor for the substrate.

In this second case study, we considered a much more complex biological dynamic system. Particularly, we studied the reaction mechanism of the irreversible inhibition of HIV proteinase, as originally examined by Kuzmic [33] (Figure 7). Note that this dynamic model has lack of practical identifiability, as reported in Rodriguez-Fernandez *et al*[4]. Nevertheless, we think that this example is still useful for the purpose of our analysis, since the emphasis here is placed on globally optimizing dynamic models of biological systems rather than analyzing identifiability issues.

The model can be described mathematically through a set of 9 nonlinear ODE’s with ten parameters:

(48) $$\frac{d[M]}{\mathrm{dt}}=-2k_{11}[M][M]+2k_{12}[E]$$

(49) $$\frac{d[P]}{\mathrm{dt}}=k_{3}[\mathrm{ES}]-2k_{41}[P][E]+2k_{42}[\mathrm{EP}]$$

(50) $$\frac{d[S]}{\mathrm{dt}}=-k_{21}[S][E]+k_{22}[\mathrm{ES}]$$

(51) $$\frac{d[I]}{\mathrm{dt}}=-k_{51}[I][E]+k_{52}[\mathrm{EI}]$$

(52) $$\frac{d[\mathrm{ES}]}{\mathrm{dt}}=k_{21}[S][E]-k_{22}[\mathrm{ES}]-k_{3}[\mathrm{ES}]$$

(53) $$\frac{d[\mathrm{EP}]}{\mathrm{dt}}=k_{41}[P][E]-k_{42}[\mathrm{EP}]$$

(54) $$\begin{array}{l} \frac{d[E]}{\mathrm{dt}}=k_{11}[M][M]-k_{12}[E]-k_{21}[S][E]+k_{22}[\mathrm{ES}]\\ \;+k_{3}[\mathrm{ES}]-k_{41}[P][E]+k_{42}[\mathrm{EP}]-k_{51}[I][E]\\ \;+k_{52}[\mathrm{EI}] \end{array}$$

(55) $$\frac{d[\mathrm{EI}]}{\mathrm{dt}}=k_{51}[I][E]-k_{52}[\mathrm{EI}]-k_{6}[\mathrm{EI}]$$

(56) $$\frac{d[\mathrm{EJ}]}{\mathrm{dt}}=k_{6}[\mathrm{EI}]$$

where the following initial conditions and parameters are known:

(57) $$\begin{array}{l} \;{\left[M\right]}_{0}=0{\left[P\right]}_{0}=0{\left[\mathrm{ES}\right]}_{0}=0\\ \;{\left[\mathrm{EP}\right]}_{0}=0\;{\left[\mathrm{EI}\right]}_{0}=0\;{\left[\mathrm{EJ}\right]}_{0}=0\\ {\left[I\right]}_{0}(\exp1)=0\;{\left[I\right]}_{0}(\exp2)=0.0015\\ {\left[I\right]}_{0}(\exp3)=0.003\;{\left[I\right]}_{0}(\exp4)=0.004\\ {\left[I\right]}_{0}(\exp5)=0.004 \end{array}$$

(58) $$\begin{array}{l} k_{11}=0.1\;k_{12}=0.001\;k_{41}=100\\ k_{21}=100\;k_{51}=100 \end{array}$$

(59) t∈[0,3600]

A series of five experiments where the enzyme HIV proteinase (E) (assay concentration 0.004 *μ*M) was added to a solution of an irreversible inhibitor (I) and a fluorogenic substrate (S) (25 *μ*M) were considered. The five experiments were carried out at four different concentrations of the inhibitor (0, 0.0015, 0.003, and 0.004 *μ*M in replicate).

The fluorescence changes were monitored during one hour. The measured signal is a linear function of the product (P) concentration, as expressed in the following equation:

(60) signal=ϵ[P]+offset

In this fit, the offset (baseline) of the fluorimeter was considered as a degree of freedom. A certain degree of uncertainty (±50%) was assumed for the value of the initial concentrations of substrate and enzyme (titration errors).

The calibration of a total of 20 adjustable parameters was addressed: five rate constants, five initial concentrations of enzyme and substrate and five offset values. Mendes and Kell [34] solved this problem using simulated annealing and reported its first known solution. Later, Rodriguez-Fernandez et al. [4] improved that solution by means of a scatter search metaheuristic, which required a fraction of the time employed by Mendes’ simulated annealing. Recall that, despite producing near optimal solutions in short CPU times, stochastic algorithms provide no information on the quality of the solutions found and are unable to guarantee convergence to the global optimum in a finite number of iterations. On the contrary, the proposed methodology ensures the global optimality of the solution computed within a desired tolerance.

In our study, the state variables were approximated using five orthogonal collocation points and five equal-length finite elements. In this case, the nonconvexities arise from the bilinear terms *ξ*_*e*, *k*, *j*_*ξ*_*e*, *k*, *j*_ and *θ*_*i*_*ξ*_*e*, *k*, *j*_.

The parameter bounds *θ*_*i*_ were set to *θ*_*i*_∈[0, 10^6^]. The lower and upper limits for the collocation coefficients *ξ*_*e*, *k*, *j*, *n*_ were fixed to *ξ*_*e*, *k*, *j*, *n*_∈[0, 37.5] except for *ξ*_*e*, *k*, E, *n*_∈[0.002, 0.006] and *ξ*_*e*, *k*, S, *n*_∈[12.5,37.5]. The bounds for all the offsets were set to offset_*n*_∈[−0.5, 0.5].

The master problem was further tightened by adding a special type of strengthening cuts. These cuts are generated by temporally decomposing the original full space MILP into a series of MILPs in each of which we fit only a subset of the original dataset, and remove the continuity equations corresponding to the extreme elements included in the sub-problem. The cuts are expressed as inequalities added to the master problem that impose lower bounds on the error of a subset of elements for which the sub-MILPs are solved. These bounds are hence obtained from the solution of a set of MILP sub-problems that optimize the error of only a subset of elements.

This case study was solved with 3 initial piecewise intervals and 6 initial hyper-planes. Two strengthening cuts involving elements 1, 2, 3 and 4, and 2, 3, 4 and 5, respectively, were added as constrains. A tolerance of 20% was used in the calculations. Hyper-planes and piecewise terms were updated at each iteration of the algorithm. In this case, BARON failed to identify any feasible solution after 12h of CPU time.

In contrast, our algorithm was able to obtain the global optimum (Table 3) with a gap of 18.64% in approximately 4,000 CPU s (Table 4). Remarkably, the solution found by our algorithm improves the best known solution reported by Rodriguez-Fernandez et al. [4]. Hence, our algorithm clearly outperformed other parameter estimation methods, improving the best known solution [4,34], and providing a rigorous lower bound on the minimum error that can be attained.

**Table 3:** Optimal parameters for the HIV proteinase inhibition problem

**Parameter**	**Rodriguez-Fernandez et al.**	**Proposed algorithm**
Sum of squares	0.01997	0.01961
k_3_ (s^−1^)	6.235	5.764
k_42_ (s^−1^)	8,772	968.7
k_22_ (s^−1^)	473	129.9
k_52_ (s^−1^)	0.09726	0.01612
k_6_ (s^−1^)	0.01417	0.01337
S_0_ exp. 1 (*μ*M)	24.63	24.61
S_0_ exp. 2 (*μ*M)	23.32	23.4
S_0_ exp. 3 (*μ*M)	26.93	27.05
S_0_ exp. 4 (*μ*M)	13.34	13.97
S_0_ exp. 5 (*μ*M)	12.5	12.5
E_0_ exp. 1 (*μ*M)	0.005516	0.005286
E_0_ exp. 2 (*μ*M)	0.005321	0.005168
E_0_ exp. 3 (*μ*M)	0.006	0.006
E_0_ exp. 4 (*μ*M)	0.004391	0.004428
E_0_ exp. 5 (*μ*M)	0.003981	0.004105
offset exp. 1	-0.004339	-0.004234
offset exp. 2	-0.001577	-0.003478
offset exp. 3	-0.01117	-0.0142
offset exp. 4	-0.001661	-0.005177
offset exp. 5	0.007133	0.00486

**Table 4:** Global optimization results for the HIV proteinase inhibition problem

	**Rodriguez-Fernandez et al.**	**BARON**	**Proposed algorithm**
Sum of squares	0.01997	failed	0.01961
UB	-	-	0.01961
LB	-	-	0.01595
Gap (%)	-	-	18.64
Iterations	29,345	263	3
Time (CPU s)	1,294	43,200	4,351

## Conclusions

In this work, we have proposed a novel strategy for globally optimizing parameter estimation problems with embedded nonlinear dynamic systems. The method presented was tested through two challenging benchmark problems: the isomerisation of *α*-Pinene (case study 1) and the inhibition of HIV proteinase (case study 2).

The proposed algorithm identified the best known solution, which was originally reported by Rodriguez-Fernandez et al. [4], in the case of the *α*-Pinene, and improved the best known one in the HIV proteinase case study. In both cases, rigorous lower bounds were provided on the global optimum, making it possible to determine the optimality gap of the solutions found.

The method proposed produced promising results, surpassing the capabilities of BARON. Our method requires some knowledge on optimization theory as well as skills using modelling systems. Our final goal is to develop a software to automate the calculations, so our approach can be easily used by a wider community. This is a challenging task, since nonlinear models are hard to handle and typically require customized solution procedures. Particularly, nonlinear models must be initialized carefully to ensure convergence even to a local solution. In this regard, the use of an outer approximation scheme that relies on a master MILP formulation is quite appealing, since the outcome of this MILP can be used to initialize the NLP in a robust manner.

Another key point here is how to construct tight relaxations of the nonconvex terms. An efficient algorithm must exploit the problem structure to obtain high quality relaxations and therefore good bounds close to the global optimum. These relaxations can be further tightened through the addition of cutting planes or the use of customized decomposition methods. As observed, there is still much work to be done in this area, but we strongly believe that such an effort is worthy. Furthermore, recent advances in global optimization theory and software applications are paving the way to develop systematic deterministic tools for the global optimization of parameter estimation problems of increasing size. Our future work will focus on making the approach more efficient through the use of tailored cutting planes and decomposition strategies and also through the hybridization of deterministic methods with stochastic approaches.

## Competing interest

The authors declare that they have no competing interests.

## Authors’ contributions

G. G-G suggested the need for the approach and J.A. E provided the biological problems. A. M, C. P, G. G-G and L. J developed the optimization algorithms and performed the numerical analysis. All authors evaluated the results, wrote the paper and contributed to its final form.
